# Potential Target Analysis of Triptolide Based on Transcriptome-Wide m^6^A Methylome in Rheumatoid Arthritis

**DOI:** 10.3389/fphar.2022.843358

**Published:** 2022-03-25

**Authors:** Danping Fan, Bin Liu, Xiaofeng Gu, Qian Zhang, Qinbin Ye, Xiaoyu Xi, Ya Xia, Qiong Wang, Zheng Wang, Bailiang Wang, Yuan Xu, Cheng Xiao

**Affiliations:** ^1^ Institute of Clinical Medicine, China-Japan Friendship Hospital, Beijing, China; ^2^ Graduate School of Peking Union Medical College, Chinese Academy of Medical Sciences/Peking Union Medical College, Beijing, China; ^3^ Institute of Basic Research in Clinical Medicine, China Academy of Chinese Medical Sciences, Beijing, China; ^4^ Biotechnology Research Institute, Chinese Academy of Agricultural Sciences, Beijing, China; ^5^ School of Traditional Chinese Medicine, Beijing University of Chinese Medicine, Beijing, China; ^6^ Laboratory for Bone and Joint Diseases, RIKEN Center for Integrative Medical Sciences, Tokyo, Japan; ^7^ Department of Orthopaedic Surgery, China-Japan Friendship Hospital, Beijing, China; ^8^ Department of TCM Rheumatology, China-Japan Friendship Hospital, Beijing, China; ^9^ Department of Emergency, China-Japan Friendship Hospital, Beijing, China

**Keywords:** triptolide, potential target, rheumatoid arthritis, m^6^A, epigenetics

## Abstract

Triptolide (TP), a major active component of the herb *Tripterygium wilfordii* Hook F (TwHF), has been shown to exert therapeutic potential against rheumatoid arthritis (RA). However, its molecular mechanism of action has not been fully elucidated. This study aimed to analyze the potential target of TP based on the discovery of differentially methylated and expressed genes (DMEGs) in RA using methylated RNA immunoprecipitation sequencing (MeRIP-seq) and RNA sequencing (RNA-seq). Five RA samples and ten control samples were obtained from China-Japan Friendship Hospital. The various levels of m^6^A methylation and genes expressed in the RA and control groups were compared by MeRIP-seq and RNA-seq. Bioinformatics explorations were also performed to explore the enriched biological roles and paths of the differentially expressed m^6^A methylation and genes. Molecular networks between TP target proteins and DMEGs were performed using Ingenuity Pathway Analysis (IPA) software. Potential target of TP was determined with Gene Expression Omnibus (GEO) database mining, molecular docking, and *in vitro* experiment validation. In total, 583 dysregulated m^6^A peaks, of which 295 were greatly upregulated and 288 were greatly downregulated, were identified. Similarly, 1,570 differentially expressed genes were identified by RNA-seq, including 539 upregulated and 1,031 downregulated genes. According to the deeper joint exploration, the m^6^A methylation and mRNA expression degrees of 35 genes varied greatly. Molecular networks between TP target proteins and DMEGs were constructed, and the results revealed that tubulin beta-2A chain (TUBB2A), insulin-like growth factor 2 mRNA-binding protein 3 (IGF2BP3), cytoplasmic dynein 1 intermediate chain 1 (DYNC1I1), and FOS-like 1 (FOSL1) were the most relevant genes that correlated with the target proteins of TP. The results of the GEO database showed that the gene expression of IGF2BP3 was increased in RA synovial tissue and consistent with the trend of our sequencing results of RA PBMCs. Molecular docking and *in vitro* experiment suggested that TP and IGF2BP3 had a high binding affinity and TP could decrease the mRNA expression of IGF2BP3 in PBMCs and MH7A.This research established a transcriptional map of m^6^A in RA PBMCs and displayed the hidden association between RNA methylation alterations and associated genes in RA. IGF2BP3 might be a potential therapeutic target of TP during RA treatment.

## Introduction

As an autoimmune disease, rheumatoid arthritis (RA) has the characteristics such as chronic inflammatory variations in synovial tissue, bone, and joint cartilage, which lead to the substantial destruction of affected joints (Cross et al., 2014; Scherer et al., 2020). The etiology of RA has not been explained completely, but the pathogenesis of RA relies on the complicated interplay between genetic and environmental triggers (Doody et al., 2017). Modern genetic technologies used in large well-characterized clinical studies have determined the participation of genetic elements in the development of RA and have advanced our understanding of the genetics of the disease (Smolen et al., 2016). However, there is no genetic heterogeneity to interpret all the characteristics of RA. Previous studies have confirmed that RA is greatly dependent on age (Sparks et al., 2016), microbiome composition (Zhang et al., 2015), certain early life factors, such as birth weight (Jacobsson et al., 2003) and breastfeeding (Karlson et al., 2004), and environmental elements, including smoking (Pedersen et al., 2007; Sparks et al., 2016). An increasing amount of evidence suggests that epigenetics contributes to the pathogenesis of RA by bridging the environmental and genetic effects. Thus, epigenetic factors are another promising area for investigating the etiology of RA.

As an epigenetic regulatory mechanism, *N* ^6^-methyladenosine (m^6^A) is the most abundant internal alteration of mRNA in eukaryotes. This alternation is reversible and regulated by “writers,” “erasers,” and “readers” (Fu et al., 2014). Recently, according to accumulating evidence, it has been reported that the m^6^A modification is indispensable for the biogenesis and roles of RNA and plays an important role in various disease pathological processes, including obesity (Loos and Yeo, 2014) and systemic lupus erythematosus (SLE) (Li et al., 2018; Luo et al. (2020b)). Many technologies for quantifying m^6^A-altered transcripts have been developed to determine the phenotypes induced by deleting proteins related to m^6^A modification and ways to affect m^6^A-altered transcripts at the molecular level, such as the transcriptome-wide scale of m^6^A modifications with methylated RNA immunoprecipitation sequencing (MeRIP-seq). Luo et al. (2020a) confirmed that, in RA, decreased alkB homolog 5 (ALKBH5), fat mass and obesity-associated protein (FTO), and YTH domain-containing family member 2 (YTHDF2) expression in peripheral blood mononuclear cells (PBMCs) is a risk factor for RA, and other research has shown that methyltransferase like 3 (METTL3) expression is greatly increased in the PBMCs of RA patients (Wang et al., 2019). Nevertheless, the transcriptome-wide m^6^A methylome in RA has not been fully characterized, and more in-depth research is rare.

*Tripterygium wilfordii* Hook F (TwHF), a traditional Chinese medicine, has been used for centuries to treat autoimmune diseases including RA in China. Previous studies have confirmed the effectiveness of TwHF in the treatment of RA (Lv et al., 2015; Zhou et al., 2018). Extracts of TwHF have also been tested in RA patients with good efficacy noted in the West (Tao et al., 2002; Goldbach-Mansky et al., 2009). As the main active ingredient in TwHF, increasing experimental evidence has verified triptolide’s (TP) anti-RA effect (Fan et al., 2016; Wang et al., 2018), and it has been considered as a promising anti-RA drug (Han et al., 2012). However, due to the complexity of disease, the underlying molecular mechanism of TP is not yet clear.

In this study, MeRIP-seq and RNA sequencing (RNA-seq) were performed, and differentially methylated peaks within mRNAs were found in RA and control samples. Meanwhile, differentially expressed mRNAs were identified using RNA-seq. In addition, further analysis of the combined MeRIP-seq and RNA-seq information was also performed for the identification of differentially methylated and expressed genes (DMEGs). Furthermore, the molecular network between TP target proteins and DMEGs was constructed using Ingenuity Pathway Analysis (IPA) software. A new potential target of TP was determined with Gene Expression Omnibus (GEO) database mining, molecular docking, and *in vitro* experiment validation.

## Materials and Methods

### Patients and Controls

Five patients suffering from RA were obtained from China-Japan Friendship Hospital. All patients had been diagnosed with RA according to the 2010 American College of Rheumatology/European League Against Rheumatism (ACR/EULAR) standards (Aletaha et al., 2010). In this study, the EULAR disease activity score (DAS28) (Prevoo et al., 1995) of five patients varied from 3.16 to 5.70. The patients were 52.00 (±9.08) years old on average. Ten subjects without RA or other autoimmune diseases were enrolled, in which five control samples (49.00 ± 7.62 years old on average) were used for MeRIP-seq, and the other five control samples (48.40 ± 5.46 years old on average) were used for the other experiments. The ethical committee of China-Japan Friendship Hospital approved the research with the ethical approval number 2020-133-K86.

### PBMC Preparation and Total RNA Extraction

PBMCs from each subject were separated using Lymphoprep™ with a density of 1.077 ± 0.001 g/ml, according to the manufacturer’s instructions (Stemcell Technologies, Canada). Total RNA in PBMCs was extracted, purified with TRIzol (Invitrogen, Carlsbad, United States) according to the manufacturer’s instructions, and stored at −80°C.

### MeRIP-Seq Assays

A NanoDrop ND-1000 (NanoDrop, Wilmington, United States) was used to quantify the total RNA amount and purity of every sample. A Bioanalyzer 2100 (Agilent, CA, United States) with an RIN > 7.0 was employed to assess the RNA integrity, followed by confirmation by electrophoresis in a denaturing agarose gel. Ribosomal RNA was depleted from approximately more than 25 μg of total RNA from a specific adipose type, based on the instructions of the Epicenter Ribo-Zero Gold Kit (Illumina, San Diego, United States). After purification, the 7-min fragmentation of ribosomal-depleted RNA into small pieces was performed with a magnesium RNA fragmentation module (NEB, United States) at 86°C. Next, the 2 h incubation of the cleaved RNA fragments was performed at 4°C with the provided m^6^A antibody (Synaptic Systems, Germany) in IP buffer (50 mM Tris-HCl, 750 mM NaCl, and 0.5% Igepal CA-630). Next, SuperScript™ II reverse transcriptase (Invitrogen) was used to reverse-transcribe the IP RNA for the creation of cDNA, which was then used to synthesize U-labeled second-stranded DNAs with *E. coli* DNA polymerase I (NEB, United States), RNase H (NEB, United States), and dUTP solution (Thermo Fisher, United States). An A-base was placed on the blunt ends of every strand for ligation to the indexed adapters. There was a T-base overhang for ligating the adapter to the A-tailed-fragmented DNA on each adapter. During the ligation of single- or dual-index adapters to the fragments, size was selected with AMPureXP beads. After treatment of the U-labeled second-stranded DNAs with the heat-labile UDG enzyme (NEB, United States), amplification of the ligated products was performed with PCR with 3-min initial denaturation at 95°C, eight cycles of denaturation at 98°C for 15 s, 15 s annealing at 60°C, 30 s extension at 72°C, and 5 min final extension at 72°C. The final cDNA library showed an average insert size of 300 ± 50 bp. Eventually, 2 × 150 bp paired-end sequencing (PE150) was performed on an Illumina Novaseq™ 6000 (LC-Bio Technology Co., Ltd., Hangzhou, China), according to the vendor’s suggested instructions (Dominissini et al., 2013).

### Data Analysis

The reads with adapter contamination, low quality bases, and uncalled bases with default coefficients were removed with fastp software (Chen et al., 2018). Then, fastp was used to verify the sequence quality of the IP and input samples. Reads were mapped to the reference genome *Homo sapiens* (Version: v101) with HISAT2 (Kim et al., 2015). The mapped reads of IP and input libraries were assessed by R package exomePeak (Meng et al., 2014) for identifying m^6^A peaks with a bed or bigwig format that can be changed for visualization on IGV software. *De novo* and known motif finding used MEME and HOMER according to localization of the motif in terms of peak summit (Bailey et al., 2009; Heinz et al., 2010). R package ChIPseeker was employed to annotate the called peaks by intersection with the gene architecture (Yu et al., 2015). Then, the expression level was determined with StringTie for all mRNAs from input libraries by using the calculation of FPKM [total exon fragments/mapped reads (millions) × exon length (kB)] (Pertea et al., 2015). A log2 (fold change) > 1 or log2 (fold change) <-1 and *p* value <0.05 by R package edgeR were used to select the differentially expressed mRNAs (Robinson et al., 2010).

### Gene Ontology and Kyoto Encyclopedia of Genes and Genomes Analysis

GO and KEGG pathway analyses were conducted on m^6^A peaks and mRNAs. GO exploration was performed, which included biological processes, cellular components, and molecular functions. On the basis of the KEGG database, the potential functions were analyzed by pathway analysis.

### Venn Analysis

Overlapped dysregulated mRNAs between the RA and control (Con) groups detected by RNA-Seq were characterized by Venn analysis. Various colors in the pie diagram show the number of mRNAs with or without overlapping.

### Analysis of Molecular Networks Between TP and DMEGs

By using “triptolide” as a key word, we searched for the potential human target proteins of TP in the PubChem platform (http://pubchem.ncbi.hlm.nih.gov). Then, the human target proteins and DMEGs were imported into the Ingenuity Pathway Analysis (IPA) platform, and molecular networks were built. In the molecular networks, nodes represent molecules, and an edge (line) shows the biological association between two nodes. At least, one reference from a textbook, literature, or canonical data saved in the IPKB supported all edges. Nodes with various shapes represent the functional class of the gene goods.

### Microarray Data

The gene expression data of GSE55235, GSE55457, and GSE55584 based on the GPL96 platform, and of GSE36700 based on the GPL570 platform were downloaded from the GEO database. The datasets contained varying number of synovial tissue samples and were selected for subsequent analysis.

### Molecular Docking

The TP’s structure was obtained from the PubChem platform (Kim et al., 2021). The PDB file for the insulin-like growth factor 2 mRNA-binding protein 3 (IGF2BP3) protein’s crystal structure was downloaded from the RCSB Protein Data Bank (PDB) database (Burley et al., 2021). Preparing and preprocessing of TP and IGF2BP3 protein were performed using AtuoDock Tools (Tanchuk et al., 2016) according to the tutorial and manual (http://vina.scripps.edu/manual.html). TP was docked with IGF2BP3 protein by AutoDock Vina (Trott and Olson, 2010). Generally, the docking score less than -5 kcal/mol was considered to have a strong binding affinity of a compound-target pair. PyMol was used for visualizing the results of AutoDock Vina (Alexander et al., 2011).

### Cell Culture

The isolated human PBMCs of the control group were cultured in RPMI 1640 medium (GIBCO, Gaithersburg, United States) and fibroblast-like synoviocytes (FLSs) cell line MH7A (JENNIO Biological Technology, Guangzhou, China) were cultured in DMEM medium (GIBCO) with 10% fetal bovine serum (GIBCO) and 1% penicillin–streptomycin in an incubator with 5% CO_2_ at 37°C.

### Treatment of PBMCs and MH7A With TP

PBMCs or MH7A were placed into 6-well plates at a concentration of 1.0 × 10^6^ cells/ml and 1 × 10^5^ cells/ml, respectively, followed by 2 h of pretreatment with or without 100 ng/ml of lipopolysaccharides (LPS) (Sigma, St. Louis, MO, United States) or 20 ng/ml recombinant human tumor necrosis factor (TNF)-α (PeproTech, New Jersey, United States). Then, PBMCs were incubated with 6.25 nM or 12.5 nM TP (purity: 99.8%, National Institutes for Food and Drug Control, Beijing, China) for another 24-h, MH7A was incubated with 12.5 nM or 25 nM TP for another 24-h. Finally, the cells were harvested for real-time PCR analysis.

### Quantitative Real-Time PCR

The PrimeScriptTM RT Reagent Kit with gDNA Eraser (Takara, Dalian, China) was used to reverse-transcribe the extracted RNA into cDNA using the instructions provided by the manufacturer. Quantitative real-time PCR analysis was performed with TB GreenTM Premix Ex TaqTM (Takara, Dalian, China) and the QuantStudio 5 Real-Time PCR System (Applied Biosystems, Bedford, MA, United States). The relative RNA expression was quantified with the 2^−ΔΔCt^ method and β-actin was used as an endogenous control. Supplementary Table S2 shows the primers used.

### Statistical Analysis

The data are shown as the mean ± SEM. The statistical significance for differences between two groups was determined with two-tailed, unpaired Student’s *t*-*test* or one-way analysis of variance (ANOVA) according to Tukey’s *post hoc* analysis. All explorations were performed with GraphPad Prism 8.0 (La Jolla, CA, United States). A *p* < 0.05 was regarded as statistically significant.

## Results

### Overview of the m^6^A Methylation Map in Human PBMCs

**FIGURE 1:**
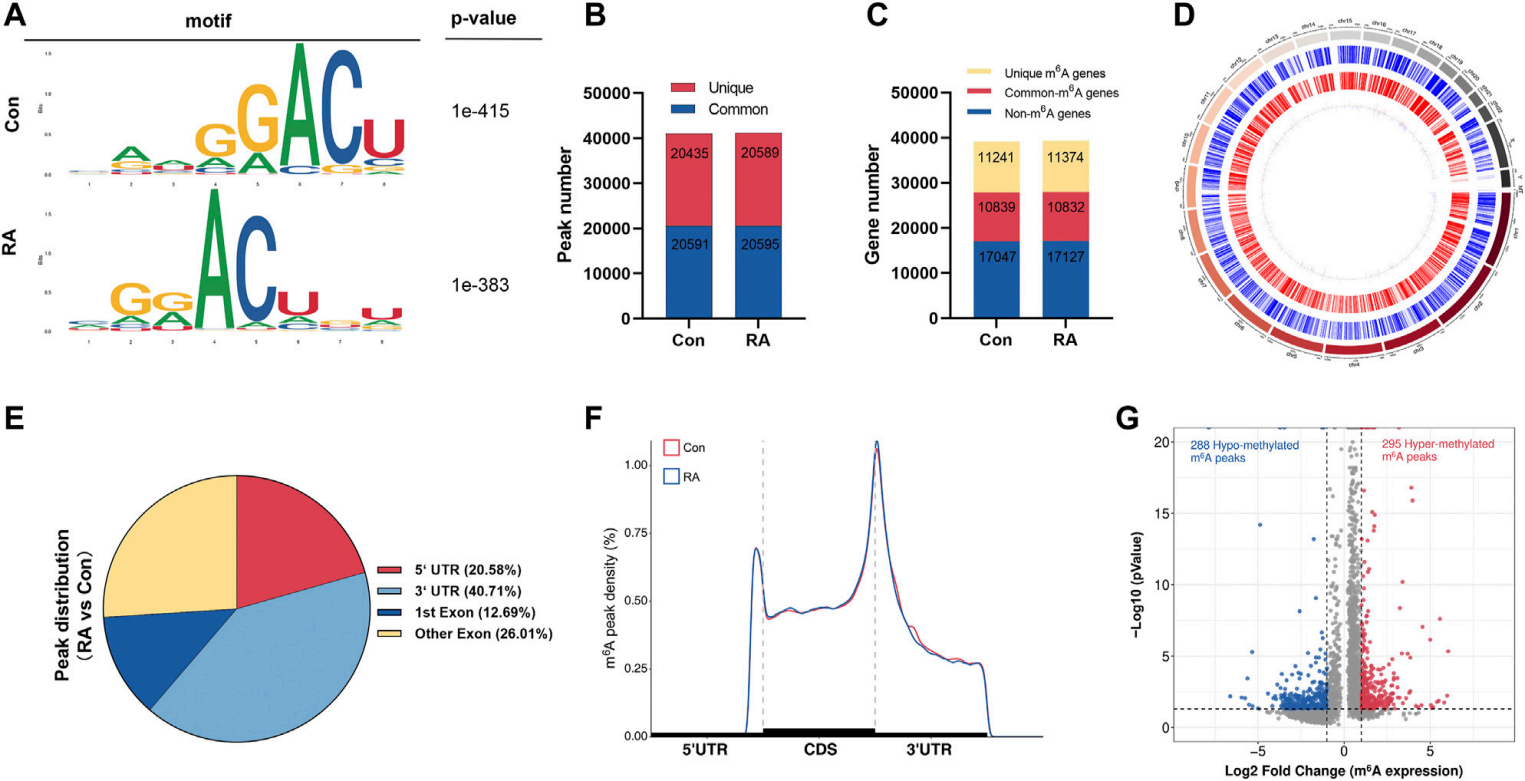
Characteristics of m^6^A methylation in RA PBMCs. **(A)** Recognition of the top consensus motif from m^6^A peaks determined in PBMCs from the RA and Con groups. **(B)** Number of m^6^A peaks recognized by m^6^A-seq in the RA and Con groups. **(C)** Summary of the m^6^A-altered genes identified using m^6^A-seq. **(D)** Distribution patterns of m^6^A peaks in various chromosomes. **(E)** m^6^A modification distribution in different gene contexts. **(F)** Accumulation of m^6^A peaks across transcripts fallen into5′-UTR, CDS, and 3′-UTR. **(G)** Recognition of the abundance of hypermethylated and hypomethylated m^6^A peaks in the RA PBMCs relative to that of the Con group. m^6^A, *N* ^*6*^-methyladenosine; RA, rheumatoid arthritis; PBMCs, peripheral blood mononuclear cells; and Con, control group.

To reveal the global m^6^A modification patterns in RA PBMCs, MeRIP-seq analysis was performed. According to Figure 1A, the m^6^A consensus motif of GGACU was presented with highly enriched in human PBMCs, which conforms to the reported consensus motif RRACH (R = G or A; H = A, C or U) (Dominissini et al., 2012). In total, 41,026 and 41,184 m^6^A peaks from 22,080 to 22,206 m^6^A-modified transcripts in Con and RA PBMCs were identified, respectively (Figures 1B,C). In contrast to the unique m^6^A peaks of the RA group (20,589) and the Con group (20,435), there were 20,595 and 20,591 common m^6^A modification peaks in the RA and Con groups, respectively (Figure 1B). In addition, 10,839 and 10,832 common m^6^A-modified genes were identified in Con and RA PBMCs, respectively, and 11,241 and 11,374 unique m^6^A-modified genes were found in Con and RA PBMCs, respectively (Figure 1C). According to the exploration of the m^6^A methylation distribution at various chromosome loci, the chromosomes with the greatest m^6^A methylation were chromosome 1 with 7,998 m^6^A methylation peaks, chromosome 17 with 5,244 m^6^A methylation peaks and chromosome 19 with 6,527 m^6^A methylation peaks (Figure 1D). The m^6^A distribution patterns within the entire transcripts were also analyzed, and the results showed a similar pattern of total m^6^A distribution in the Con and RA groups. For the dysregulated peaks, 40.71% were in the 3′-UTR area, 20.58% were in the 5′-UTR area, 12.69% were in the first exon, and 26.01% were in the residual exons (Figures 1E,F). Furthermore, the m^6^A peak abundance of common m^6^A-altered genes between PBMCs of the Con and RA patients was compared. We discovered 295 hypermethylated m^6^A peaks and 288 hypomethylated m^6^A peaks in the RA PBMCs relative to the Con PBMCs (log2 |fold change| > 1 and *p* value <0.05; Figure 1G). Table 1 shows the top 20 differentially methylated genes (DMGs), and Supplementary Table S3 displays the list of all DMGs.

**TABLE 1:** Top 20 differentially methylated m^6^A peaks (RA/Con).

Gene	Transcript ID	Chr	Start	End	FC	*p* value	m^6^A regulation
RPS29	ENST00000396020	chr14	49572271	49572331	65.80	0.00	Hyper
SPIC	ENST00000551346	chr12	101486404	101486730	56.10	0.02	Hyper
AC007952	ENST00000572818	chr17	19160997	19161294	47.18	0.00	Hyper
MAP6	ENST00000304771	chr11	75594370	75594517	44.94	0.01	Hyper
WWC3	ENST00000380861	chrX	10015284	10015493	41.93	0.02	Hyper
AC009292	ENST00000502156	chr15	67835614	67835762	36.00	0.04	Hyper
DYNC1I1	ENST00000537881	chr7	95772685	95802770	34.06	0.04	Hyper
AC007952	ENST00000572818	chr17	19161650	19161739	32.90	0.03	Hyper
GTF2H2C	ENST00000512736	chr5	69566147	69566297	32.00	0.00	Hyper
ENPP7P6	ENST00000529817	chr8	12469791	12469941	23.43	0.00	Hyper
TBC1D3I	ENST00000616671	chr17	36254080	36254347	0.02	0.01	Hypo
AL627309	ENST00000466557	chr1	164407	164737	0.02	0.00	Hypo
CCDC144A	ENST00000399273	chr17	16735543	16737617	0.02	0.03	Hypo
AP002954	ENST00000526274	chr11	118721104	118721253	0.03	0.05	Hypo
CENPBD1P1	ENST00000651608	chr19	58602200	58602380	0.05	0.00	Hypo
NBEA	ENST00000400445	chr13	35251245	35251362	0.06	0.03	Hypo
WSCD2	ENST00000547525	chr12	108248051	108248470	0.06	0.01	Hypo
NOMO3	ENST00000263012	chr16	16232588	16232828	0.06	0.01	Hypo
SLC12A8	ENST00000430155	chr3	125083622	125083950	0.07	0.00	Hypo
SNX25	ENST00000618785	chr4	185209586	185209766	0.08	0.01	Hypo

### Differentially Methylated Genes Participated in Important Biological Functions and Pathways

**FIGURE 2:**
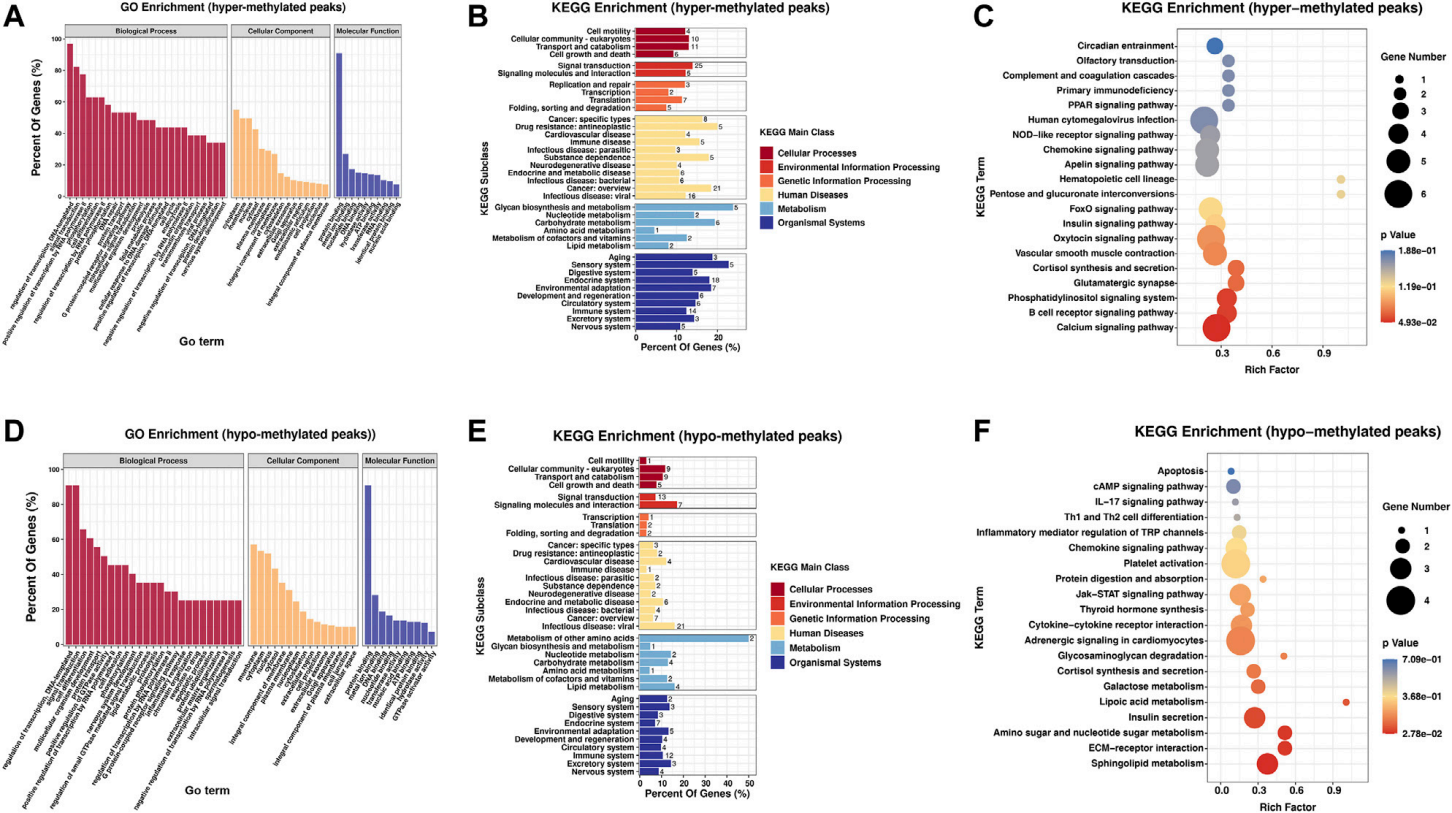
Biological function and pathway analysis of differentially methylated genes between the RA and Con groups. **(A)** GO enrichment exploration of hypermethylated peaks. **(B)** KEGG enrichment subclass analysis of hypermethylated peaks. **(C)** Top 20 KEGG pathways exploration of hypermethylated peaks. **(D)** GO enrichment exploration of hypomethylated peaks. **(E)** KEGG enrichment subclass analysis of hypomethylated peaks. **(F)** Top 20 KEGG pathways in the hypomethylated peaks. RA, rheumatoid arthritis; Con, control group; GO, Gene Ontology; and KEGG, Kyoto Encyclopedia of Genes and Genomes.

To study the biological importance of m^6^A modification in RA, GO enrichment and KEGG pathway analyses were performed. GO analysis results showed that both hypermethylated and hypomethylated genes in the PBMCs participated in the following functions and pathways: “regulation of transcription, DNA-templated,” “signal transduction,” and “cell differentiation” (ontology: biological process); “cytoplasm,” “membrane,” and “nucleus” (ontology: cellular component); and “protein binding,” “nucleotide binding,” “metal ion binding,” and “DNA binding” (ontology: molecular function) (Figures 2A,D). In KEGG subclass analyses, hypermethylated and hypomethylated genes were involved in the following functions and pathways: “cell motility,” “cellular community-eukaryotes,” and “cell growth and death” (cellular processes); and “signal transduction” and “signaling molecules and interaction” (environmental information processing). In addition, hypermethylated and hypomethylated genes were associated with “immune disease” (human diseases) and “immune system” (organismal systems) (Figures 2B,E). For KEGG pathway enrichment, we found that the hypermethylated genes were greatly associated with the “calcium signaling pathway” and the “B-cell receptor signaling pathway” (Figure 2C). However, the hypomethylated genes were significantly related to “sphingolipid metabolism” and “ECM-receptor interaction” (Figure 2F).

### Overview of the Transcriptome Profiles

**FIGURE 3:**
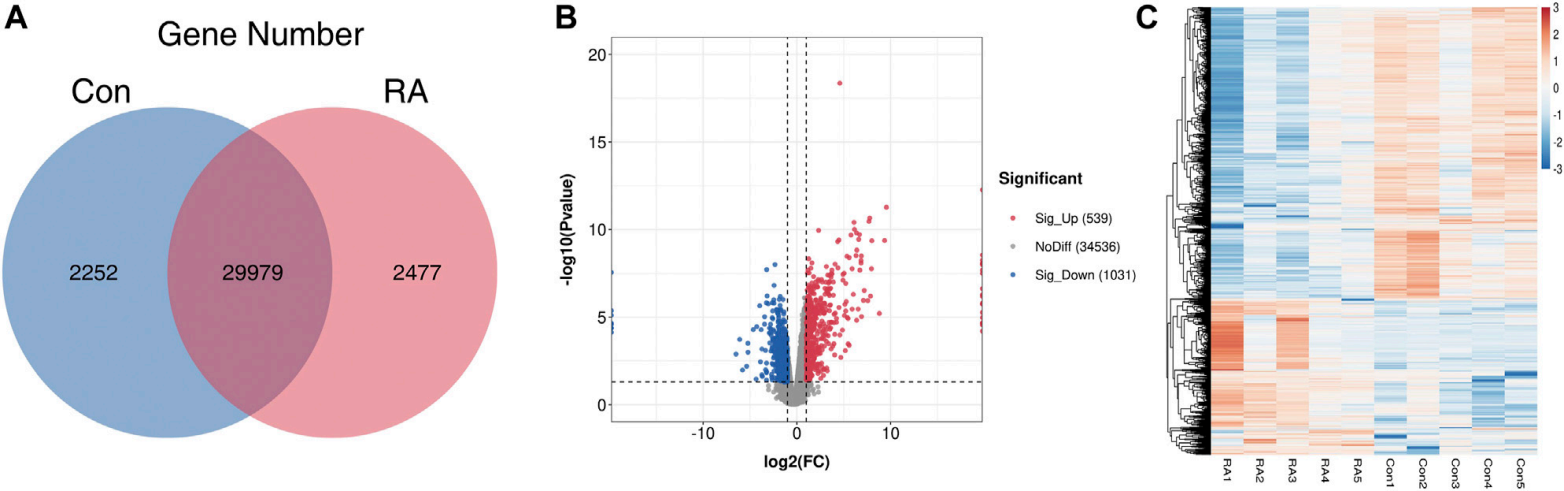
RNA-seq data analysis and the differentially expressed genes between the RA and Con groups. **(A)** Venn diagram of RNA-seq data displaying the common and unique genes between the RA and Con groups. **(B)** Volcano plots showing the differentially expressed genes in RA PBMCs compared with those in the Con group. **(C)** Heatmap plots displaying the differentially expressed genes in RA PBMCs vs. those in the Con group. RA, rheumatoid arthritis; Con, control group; and PBMCs, peripheral blood mononuclear cells.

To clarify the altered gene expression and the corresponding signaling pathways in RA PBMCs, RNA-seq was performed to identify the differentially expressed genes (DEGs). In Figure 3A, the differences and overlaps of genes in the RA and Con groups are shown using a Venn diagram. We found that 2,252 genes in the Con group, 2,477 genes in the RA group, and 29,979 genes were common between the two groups. Next, applying the standard of *p* value <0.05 and fold change (FC) ≥ 2, 1,570 genes were shown to be greatly dysregulated compared with the Con group, including 539 upregulated genes and 1,031 downregulated genes (Figure 3B). Figure 3C shows the hierarchical clustering of the DEGs. The full list of upregulated and downregulated genes is contained in Supplementary Table S4.

### Differentially Expressed Genes Participated in Important Biological Functions and Pathways

**FIGURE 4:**
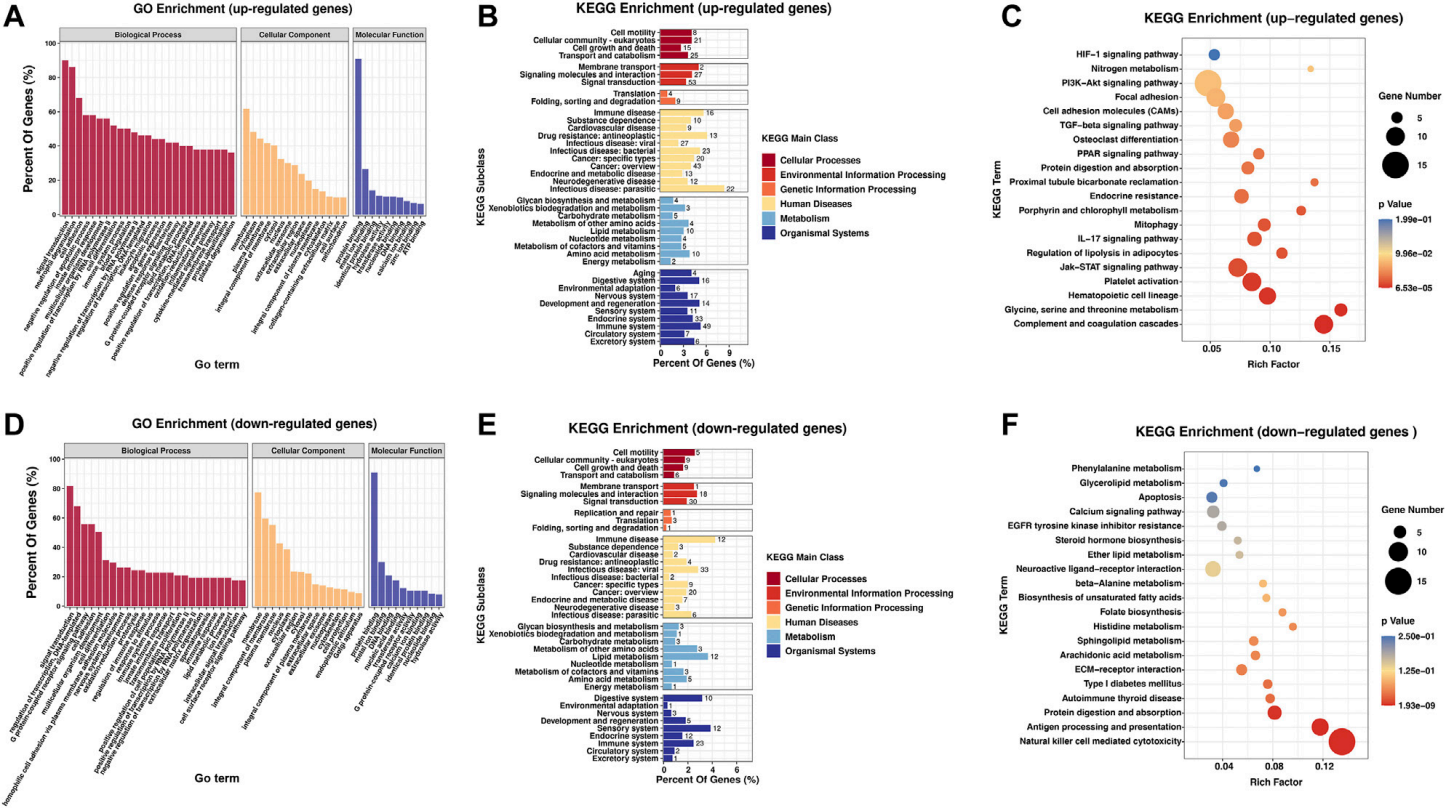
Biological function and pathway analysis of differentially regulated genes between the RA and Con groups. **(A)** GO enrichment analysis of the upregulated genes with great diversity. **(B)** KEGG enrichment subclass exploration of the upregulated genes with significant differences. **(C)** Top 20 KEGG pathway enrichments of the upregulated genes with great diversity. **(D)** GO enrichment exploration of the significantly downregulated genes. **(E)** KEGG enrichment subclass exploration of the highly downregulated genes. **(F)** Top 20 KEGG pathway enrichments of the significantly downregulated genes. RA, rheumatoid arthritis; Con, control group; GO, Gene Ontology; and KEGG, Kyoto Encyclopedia of Genes and Genomes.

To analyze the biological functions and signaling pathways involved in DEGs, GO and KEGG analyses were performed. The outcomes of the GO analysis suggested the enrichment of these genes in the following functions and pathways: “signal transduction” and “cell adhesion” (ontology: biological process); “membrane,” “cytoplasm,” and “integral component of membrane” (ontology: cellular component); and “protein binding” and “DNA binding” (ontology: molecular function). Upregulated and downregulated genes were also involved in “neutrophil degranulation” and “regulation of transcription, DNA-templated” (ontology: biological process), respectively (Figures 4A,D).

In KEGG subclass analyses, upregulated and downregulated genes were involved in the following functions and pathways: “cell motility,” “cellular community-eukaryotes,” “cell growth and death,” and “transport and catabolism” (cellular processes); and “membrane transport,” “signaling molecules and interaction,” and “signal transduction” (environmental information processing). In genetic information processing, upregulated and downregulated genes were involved in “folding, sorting, and degradation.” In metabolism, “glycan biosynthesis and metabolism” was associated with the upregulated and downregulated genes. In addition, upregulated and downregulated genes were associated with “immune disease” (human diseases) and “immune system” (organismal systems) (Figures 4B,E). According to KEGG pathway exploration, the upregulated genes were strongly related to “complement and coagulation cascades,” “Jak-STAT signaling pathway” and “IL-17 signaling pathway” (Figure 4C). However, the downregulated genes were significantly related to “natural killer cell mediated cytotoxicity,” “antigen progressing and representation,” and “protein digestion and absorption” (Figure 4F).

### Conjoint Analyses of MeRIP-Seq and RNA-Seq Data

**FIGURE 5:**
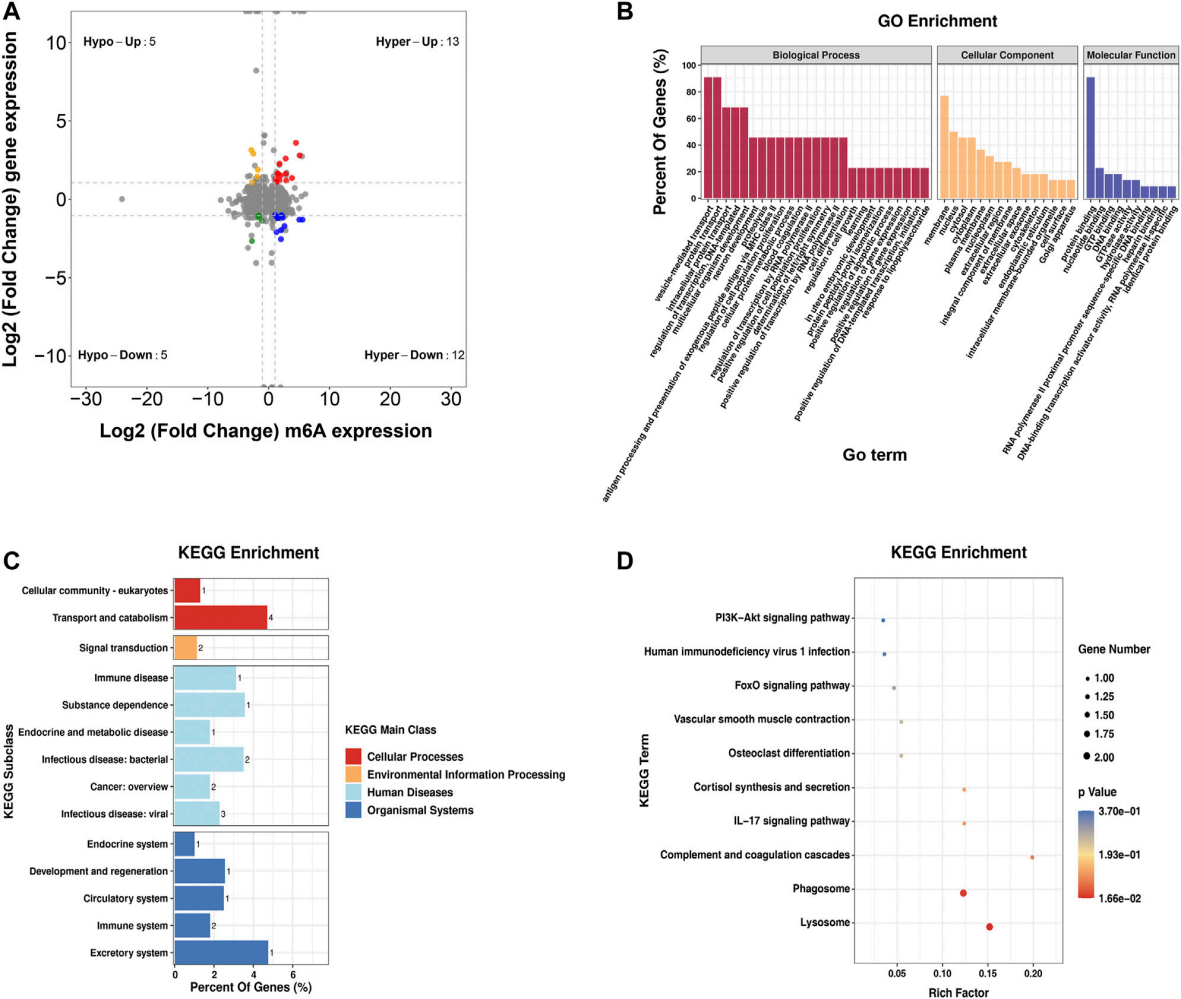
Conjoint analysis of the MeRIP-seq and RNA-seq data. **(A)** Distribution of genes with a great variation in both the m^6^A modification and mRNA levels in the PBMCs of RA relative to that of Con. **(B)** GO enrichment exploration of genes with great variation in both the m^6^A modification and mRNA levels. **(C)** KEGG subclass of genes with great variation in both the m^6^A modification and mRNA levels. **(D)** KEGG pathway enrichments of differentially expressed genes at both the m^6^A modification and mRNA levels. PBMCs, peripheral blood mononuclear cells; RA, rheumatoid arthritis; Con, control group; GO, Gene Ontology; m^6^A, N^6^-methyladenosine; and KEGG, Kyoto Encyclopedia of Genes and Genomes.

Furthermore, by the conjoint analysis of MeRIP-seq and RNA-seq data, 35 DMEGs changed significantly, including 13 hypermethylated and upregulated genes (hyper-up), 12 hypermethylated and downregulated genes (hyper-down), five hypomethylated and upregulated genes (hypo-up), and five hypomethylated and downregulated genes (hypo-down) (Figure 5A). Table 2 shows the list of 35 DMEGs. The GO analysis revealed that the DMEGs were associated with the following functions and pathways: “protein transport,” “intracellular protein transport,” and “multicellular organism development,” (biological process); “membrane,” “nucleus,” and “cytosol” (cellular component); and “protein binding,” “nucleotide binding,” and “GTP binding” (molecular function) (Figure 5B). In KEGG subclass analysis, the DMEGs were involved in “cellular community” and “transport and catabolism” (cellular processes) and “signal transduction” (environment information processing). In human diseases, DMEGs were related to “immune disease” (Figure 5C). The KEGG pathway analysis displayed the primary enrichment of these DMEGs in “lysosome,” “phagosome,” and “component and coagulation cascades” (Figure 5D).

**TABLE 2:** Differentially expressed genes with differentially methylated peaks.

Gene	Transcript ID	Chr	Start	End	m^6^A regulation	FC	*p* value	Gene regulation
TLCD4	ENST00000370203	chr1	95163982	95164072	Up	12.15	0.00	Up
TFPI	ENST00000392365	chr2	187466956	187467016	Up	2.87	0.00	Up
CALD1	ENST00000466704	chr7	134948102	134958076	Up	3.06	0.00	Up
ARL4A	ENST00000396663	chr7	12689644	12689823	Up	3.10	0.00	Up
IGF2BP3	ENST00000258729	chr7	23312036	23312275	Up	2.53	0.00	Up
DYNC1I1	ENST00000537881	chr7	95772685	95802770	Up	6.94	0.00	Up
PBX1	ENST00000420696	chr1	164682414	164682654	Up	2.55	0.00	Up
AC010615	ENST00000596705	chr19	21444241	21448107	Up	6.04	0.00	Up
RAB2B	ENST00000397762	chr14	21476870	21476960	Up	2.32	0.00	Up
LIPH	ENST00000296252	chr3	185508295	185508564	Up	2.17	0.00	Up
FOSL1	ENST00000312562	chr11	65892049	65892259	Up	4.80	0.00	Up
C1orf226	ENST00000426197	chr1	162381926	162382196	Up	3.08	0.01	Up
H3C8	ENST00000614378	chr6	26271488	26271755	Up	2.28	0.03	Up
AC120053	ENST00000607706	chr8	93715995	93716113	Up	0.17	0.00	Down
CEP290	ENST00000676363	chr12	88114472	88115512	Up	0.50	0.00	Down
FKBP2	ENST00000309366	chr11	64241095	64241508	Up	0.25	0.01	Down
AC022706	ENST00000662865	chr17	35325031	35325210	Up	0.43	0.01	Down
ZNF577	ENST00000638827	chr19	51804876	51805175	Up	0.50	0.01	Down
BAALC	ENST00000438105	chr8	103228011	103228338	Up	0.23	0.01	Down
AP1S3	ENST00000443700	chr2	223755945	223756184	Up	0.43	0.02	Down
SGK2	ENST00000373077	chr20	43584852	43585509	Up	0.30	0.02	Down
AC007952	ENST00000572818	chr17	19160997	19161294	Up	0.40	0.02	Down
AC007952	ENST00000572818	chr17	19161650	19161739	Up	0.40	0.02	Down
NAPSA	ENST00000253719	chr19	50362389	50362568	Up	0.46	0.03	Down
AL683813	ENST00000435597	chX	136840961	136841469	Up	0.44	0.04	Down
TUBB2A	ENST00000333628	chr6	3154377	3154554	Down	8.81	0.00	Up
PCSK6	ENST00000558864	chr15	101299388	101299538	Down	2.72	0.00	Up
RAB6B	ENST00000285208	chr3	133825793	133826063	Down	3.69	0.00	Up
LRRC32	ENST00000260061	chr11	76659977	76660785	Down	2.11	0.00	Up
LHFPL6	ENST00000648377	chr13	39601116	39601325	Down	7.51	0.00	Up
DISP1	ENST00000674736	chr1	222991587	222991974	Down	0.48	0.00	Down
KRT18P4	ENST00000422599	chr20	49956895	49957194	Down	0.38	0.01	Down
CRYGS	ENST00000392499	chr3	186546583	186546702	Down	0.48	0.02	Down
TENM4	ENST00000278550	chr11	78670294	78672074	Down	0.16	0.03	Down
AL683813	ENST00000435597	chX	136842664	136842843	Down	0.44	0.04	Down

### Potential Target Analysis of TP and Hub Gene Validation

**FIGURE 6:**
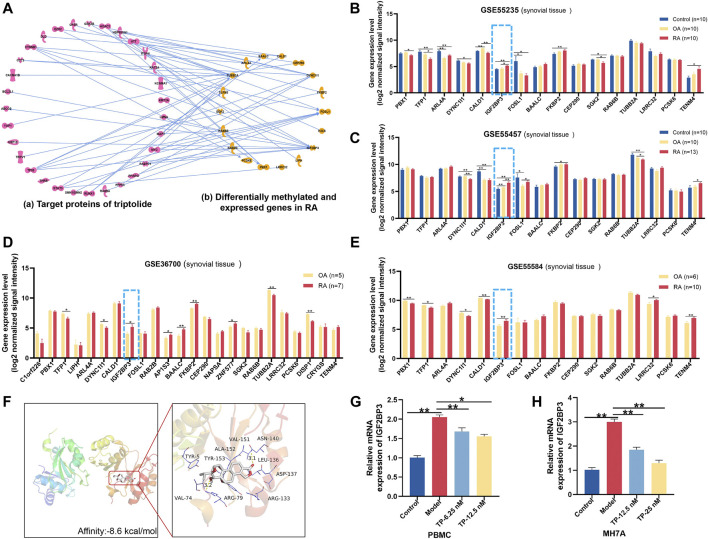
Potential target analysis of TP. **(A)** Network analysis between genes with great variation in both the m^6^A modification and mRNA levels and target proteins of TP. **(B–E)** Gene expression of IGF2BP3 in synovial tissue samples of GEO datasets. **(F)** Molecular docking of IGF2BP3 and TP. In the picture on the right, thick sticks represent the TP molecule, and thin lines represent residues in the protein binding site. **(G,H)** mRNA expression of IGF2BP3 in PBMCs and MH7A. *p < 0.05, ** *p < 0.01*. TP, triptolide; IGF2BP3, insulin-like growth factor 2 mRNA-binding protein 3; GEO, Gene Expression Omnibus; and PBMCs, peripheral blood mononuclear cells.

To construct the molecular network between TP target proteins and candidate DMEGs, we first searched and found 169 target proteins of TP (Supplementary Table S5) in the PubChem platform. Then, the molecular network of TP target proteins and DMEGs was built. The result revealed that the tubulin beta-2A chain (TUBB2A), IGF2BP3, cytoplasmic dynein 1 intermediate chain 1 (DYNC1I1), and FOS-like 1 (FOSL1) were the most relevant genes that correlated with the target proteins of TP (Figure 6A). In addition, the gene expression of IGF2BP3 in RA synovial tissue of different GEO datasets was increased (Figures 6B–E) and consistent with the trend of our sequencing results of RA PBMCs. So, we chose IGF2BP3 as a candidate gene for further analysis. To verify the binding between IGF2BP3 and TP, we performed molecular docking. The crystal structure of IGF2BP3 (PDB, ID: 6fq1) was obtained from RCSB Protein Data Bank and the chemical structure of TP was downloaded from the PubChem platform. The docking scores between IGF2BP3 and TP is -8.6 kcal/mol (Figure 6F), suggesting that TP and IGF2BP3 have a higher binding affinity. The m^6^A peak distributions of IGF2BP3 between the Con and RA groups are shown in Supplementary Figure S1A. Then, the mRNA expression of IGF2BP3 in PBMCs and MH7A were validated by RT-PCR and found to be increased, similar to our sequence data, while after treatment with different concentrations of TP, IGF2BP3 mRNA level was decreased (Figures 6G,H). These results suggested that IGF2BP3 might be a new potential target of TP during the treatment of RA.

## Discussion

RA is an autoimmune disease that is characterized by chronic inflammation and progressive bone destruction of joints (Smolen et al., 2016). Multiple studies have suggested that the interplay among immunity, lifestyle, and genetic and environmental factors plays an important role in RA pathogenesis. Recently, an increasing body of evidence has revealed that epigenetics contributes to the pathogenesis of RA, probably by integrating genetic and environmental effects (Gray, 2014; Smolen et al., 2016). m^6^A, an epigenetic regulatory mechanism, is the most abundant internal modification of mRNA in eukaryotes and modulates the gene expression program at the posttranscriptional level. Variant m^6^A levels exert various biological functions such as cell differentiation, neuronal signaling, and immune tolerance. m^6^A modification is involved in various diseases, such as obesity, diabetes, and SLE (Church et al., 2010; Shen et al., 2015; Luo et al., 2020a). A recent study showed that METTL3 can promote activation and inflammation in FLSs and the adjuvant-induced arthritis animal model using nuclear factor (NF)-κB signaling pathway (Shi et al., 2021). Another study revealed that the m^6^A modification of TGM2 mRNA contributes to the inhibitory activity of sarsasapogenin in RA FLS (Lin et al., 2022). Nevertheless, as a complex disease, the effect of m^6^A modification on RA progression remains to be clarified.

Numerous studies have shown that peripheral blood represents the main highway for the RA immune system. PBMCs are immune cells and can initiate the autoimmune inflammatory process directed against target organs. Therefore, the gene expression signatures of PBMCs can shed light on the molecular features of the immune cells in the targeted organs in RA progression (Wang et al., 2022). Our research obtained the first MeRIP-seq profile in RA PBMCs. MeRIP-seq revealed 41,026 and 41,184 m^6^A peaks from 22,080 to 22,206 m^6^A-modified transcripts in Con and RA PBMCs, respectively. We found the enrichment of the m^6^A peaks of RA and Con PBMCs in the 3′-UTR area and near the stop codon, which were conformed with the results from past studies (Dominissini et al., 2012; Meyer et al., 2012; He and He, 2021). These sites were m^6^A-specific. Compared with the Con group, 295 hypermethylated m^6^A peaks and 288 hypomethylated m^6^A peaks in the RA PBMCs were identified. These differentially methylated peaks were greatly enriched in “signal transduction,” “cell differentiation,” “regulation of transcription,” “multicellular organism development,” “cell motility,” and “cellular community-eukaryotes,” suggesting the conserve and basic effects of m^6^A in controlling growth and cell fate specification (Zaccara et al., 2019). In KEGG pathway enrichment, it was observed that upregulated m^6^A modification genes are associated with the “calcium signaling pathway” and the “B-cell receptor signaling pathway.” Calcium (Ca^2+^) signaling is a common hub for many signaling pathways that take part in tolerance mechanisms, and B cells express a large panel of ion channels and transporters that contribute to the production and regulation of Ca^2+^ signals, which exert a significant effect on B-cell growth and fate (Hemon et al., 2017). Signaling by intracellular Ca^2+^ has been shown to be involved in RA pathogenesis. Autoreactive B cells also exert a key effect on the pathogenesis of RA. Defects in Ca^2+^ influx kinetics and enhancement of basal [Ca^2+^]_cyt_ have been found in T lymphocytes isolated from RA patients (Toldi et al., 2013). These studies suggested that m^6^A modification may boost the appearance and progression of RA by controlling the calcium signaling pathway or the B-cell receptor signaling pathway. Interestingly, the hypomethylated genes were significantly related to “sphingolipid metabolism.” A previous study reported that sphingolipids are a category of complicated lipids with a backbone of sphingoid bases and that several bioactive sphingolipids are involved in the pathological processes of RA (Gomez-Larrauri et al., 2020), which indicates that m^6^A modification may affect RA progression by regulating sphingolipid metabolism. However, the precise functions of sphingolipid metabolism in RA still need to be studied in-depth, and the m^6^A-altered genes associated with this pathway in RA must be further clarified.

Next, through RNA-seq analyses, 1,570 significantly dysregulated genes were identified, including 539 upregulated genes and 1,031 downregulated genes. These dysregulated genes were significantly involved in “signal transduction,” “cell adhesion” and “neutrophil degranulation.” In KEGG pathway analyses, several canonical pathways associated with RA inflammation were significantly enriched, including “complement and coagulation cascades,” “Jak-STAT signaling pathway” and “IL-17 signaling pathway.” However, the downregulated genes were significantly related to “natural killer cell mediated cytotoxicity.”

To elucidate the mechanism of m^6^A modification in influencing RA development, conjoint explorations of MeRIP-seq and RNA-seq data were performed, and 35 DMEGs were found. The enrichment of genes in “protein transport” and “protein binding” was observed, and KEGG pathway analysiss howed that these DMEGs were enriched primarily in “lysosome.” Lysosomes are membrane-bound organelles involved in the processes of degrading and recycling cellular waste, cellular signaling, and energy metabolism (Bonam et al., 2019). Increasing evidence implicates the effects of lysosomal dysfunction in more common diseases such as inflammatory and autoimmune disorders, including RA. Weissmann G concluded that various cathepsins, acid phosphatases, and other enzymes in humans participate in inflammation and joint damage (Weissmann, 1966). The earlier results indicated that the “lysosome” function in which DMEGs are involved might play an important role in RA pathogenesis and might be a therapeutic target.

TP is the main active ingredient extracted from TwHF, and it has been considered a promising anti-RA drug (Fan et al., 2018), but the underlying molecular mechanism of TP on RA has still not been fully elucidated. To better discover potential targets of TP and elucidate the molecular mechanism of TP in our further study, we chose TP as a therapeutic candidate and constructed the molecular network of TP target proteins and candidate DMEGs obtained from our MeRIP-seq and RNA-seq results. Our results showed that there was a close relationship between the target proteins of TP and the DMEGs of RA. The TUBB2A, IGF2BP3, DYNC1I1, and FOSL1 had high-degree nodes with the target proteins of TP in the network, suggesting that they might exert key effects on the response to TP. Considering the critical role of synovial and FLSs in RA (Nygaard and Firestein, 2020), we searched the GEO database and found four different datasets of RA synovial tissue in which the gene expression of IGF2BP3 was increased. This result was consistent with the trend of our sequencing results of RA PBMCs and suggested that IGF2BP3 might be an important virulence gene in RA.

IGF2BP3 is a highly conserved paralog of IGF2BPs and primarily exerts oncogenic effects in cancer (Mancarella and Scotlandi, 2019). In recent years, some studies have increasingly recorded the effects of IGF2BP3 on basic processes in cancer biology, and its overexpression has been extensively related to adverse patient results in various tumors, such as colon cancer and gastric carcinogenesis (Zhou et al., 2017; Yang et al., 2020). However, studies on IGF2BP3 in RA are rare. In our results, the docking scores between IGF2BP3 and TP is -8.6 kcal/mol, indicating that TP and IGF2BP3 have a higher binding affinity. The mRNA expression of IGF2BP3 in PBMCs and MH7A was increased, similar to our sequence data, while after treatment with different concentrations of TP, IGF2BP3 mRNA level was decreased, demonstrating that IGF2BP3 might be a new target of TP, which provide a possible strategy for the treatment of RA. Of course, elucidating the fundamental mechanisms by which m^6^A and TP regulate gene expression is an important direction of our future studies.

## Conclusion

This study established a transcriptional map of m^6^A in RA PBMCs and displayed the hidden association between m^6^A methylation modification and associated genes in RA. The results highlight the importance of m^6^A modification as a gene regulatory system in RA. IGF2BP3 might be a potential therapeutic target of TP during the treatment of RA. More research based on these epigenetic clues should be conducted in the future to deepen the understanding of the mechanisms of TP and m^6^A modification in RA.

## Data Availability

The datasets presented in this study can be found in online repositories. The names of the repository/repositories and accession number(s) can be found below: National Center for Biotechnology Information (NCBI) BioProject database under accession number GSE193193.
