# Supplementary material for: Potential Target Analysis of Triptolide Based on Transcriptome-Wide m6A Methylome in Rheumatoid Arthritis
Source: Front Pharmacol. 2022 Mar 25;13:843358. doi: 10.3389/fphar.2022.843358 (PMC8993230; doi:10.3389/fphar.2022.843358)
Supplement: Supplementary file 6 [file DataSheet1.docx]

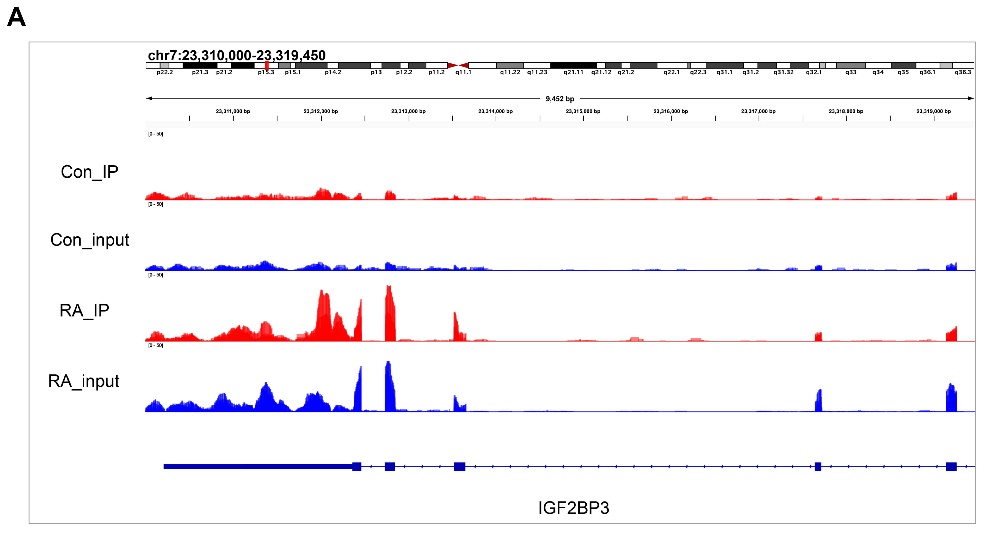


**Figure S1.** The RNA (input) and m^6^A (IP) abundance in IGF2BP3 mRNA transcripts in the Con and RA groups as detected by RNA-seq and MeRIP-seq.


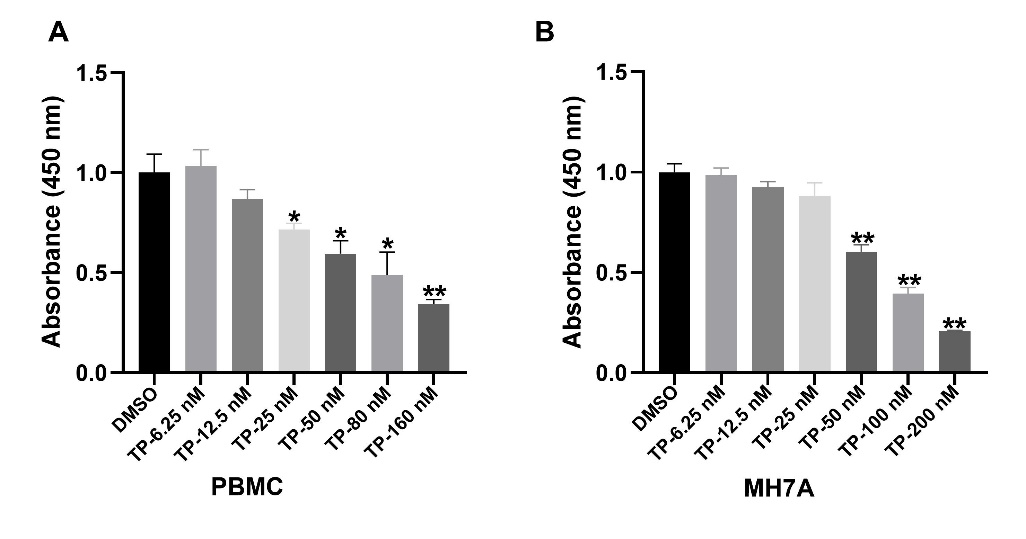


**Figure S2.** Cytotoxicity of triptolide (TP) on peripheral blood mononuclear cells (PBMCs) and MH7A cell line. PBMCs and MH7A were treated with different concentration of TP for 24 h, and their viability was determined using cell counting kit (CCK)-8 assay. ^**^ *p < 0.01*, ^*^ *p < 0.05 versus* group without TP treatment (DMSO group). The data are presented as the mean ± SEM from three independent experiments.
